# Survey and Experimental Infection of Enteropathogenic *Escherichia coli* in Common Marmosets (*Callithrix jacchus*)

**DOI:** 10.1371/journal.pone.0160116

**Published:** 2016-08-08

**Authors:** Nobuhito Hayashimoto, Takashi Inoue, Hanako Morita, Masahiko Yasuda, Masami Ueno, Kenji Kawai, Toshio Itoh

**Affiliations:** 1 ICLAS Monitoring Center, Central Institute for Experimental Animals, 3-25-12 Tonomachi, Kawasaki-ku, Kawasaki, Japan; 2 Marmoset Research Department, Central Institute for Experimental Animals, 3-25-12 Tonomachi, Kawasaki-ku, Kawasaki, Japan; 3 Pathological Analysis Center, Central Institute for Experimental Animals, 3-25-12 Tonomachi, Kawasaki-ku, Kawasaki, Japan; Tulane University, UNITED STATES

## Abstract

Common marmosets (*Callithrix jacchus*) are frequently used for biomedical research but can be afflicted with diarrhea—a serious and potentially lethal health problem. Enteropathogenic *Escherichia coli* (EPEC) is thought to be the causative pathogen of hemorrhagic typhlocolitis in common marmosets, but the actual incidence of the disease and the relationship between EPEC and hematochezia are unknown. This study investigated the prevalence of EPEC infection in common marmosets and the association between EPEC and hematochezia. A total of 230 stool or rectal swab samples were collected from 230 common marmosets (98 clinically healthy, 85 diarrhea, and 47 bloody stool samples) and tested by culture-based detection and PCR amplification of VT1, VT2, LT, ST, *eae*, and *bfp* genes. Healthy animals were divided into three groups (n = 4 each for high and low concentration groups and n = 2 as negative control), and those in the experimental groups were perorally inoculated with a 2-ml of suspension of EPEC R811 strain adjusted to 5 × 10^8^ (high concentration) and 5 × 10^4^ (low concentration) CFU/ ml. Two animals in each group were examined 3 and 14 days post-inoculation (DPI). EPEC was detected in 10 of 98 clinically healthy samples (10.2%), 17 of 85 diarrhea samples (20%), and all 47 bloody stool samples (100%), with a significant difference detected between presence of EPEC and sample status (P < 0.01). Acute hematochezia was observed in all animals of the high-concentration group but not in other groups at 1 or 2 DPI. A histopathological examination revealed the attachment of gram-negative bacilli to epithelial apical membranes and desquamated epithelial cells in the cecum of animals in the high-concentration group at 3 DPI. These findings suggest that EPEC is a causative agent of hemorrhagic typhlocolitis in common marmosets.

## Introduction

The common marmoset (*Callithrix jacchus*) is a New World primate used for biomedical research [1]. Diarrhea is a significant health problem in laboratory-bred common marmosets that occasionally leads to death.

Several intestinal pathogens cause diarrhea in common marmosets [2,3]. Enteropathogenic *Escherichia coli* (EPEC) is thought to be the causative pathogen of hemorrhagic typhlocolitis [4]. EPEC is regarded as an important cause of chronic diarrhea in humans, especially in children [5,6] and can produce attaching and effacing lesions that are characterized by tight attachment of bacteria to the enterocyte surface, effacement of brush border microvilli, and formation of an actin-rich pedestal beneath attached bacteria [5,6].

Only a few methods such as survey and necropsy have been used to investigate hemorrhagic typhlocolitis associated with EPEC in common marmosets [4], and the actual incidence of this disease remains unknown. To address this issue, we carried out a survey of EPEC in fecal and rectum samples of common marmosets in various conditions (bloody stools, diarrhea without blood, and clinically healthy) to investigate the association between EPEC and hematochezia. In addition, marmosets were experimentally infected with EPEC isolates to characterize EPEC infection in these animals.

## Materials and Methods

### Survey of EPEC in fecal and rectal swab samples

#### Samples

A total of 230 stool and rectal swab samples were collected from 230 common marmosets (92 males and 138 females) kept in the Central Institute for Experimental Animals (CIEA) between June 2011 and August 2012. The animals were obtained a commercial vendor (CLEA Japan, Tokyo, Japan) or were born at the CIEA. The animals used in the study ranged in age from 1 month to 14.2 years (males: 2 months–14.2 years; females: 1 month–12.1 years) were housed in a conventional indoor facility in accordance with the Regulations for Animal Experimentation of CIEA based on the Guidelines for Proper Conduct of Animal Experiments (Science Council of Japan, 2006), and were negative for *Salmonella* spp., *Shigella* spp., and *Yersinia pseudotuberculosis* in yearly monitoring. There were 98 healthy stool or rectal samples (clinically healthy samples), 85 diarrhea or rectal swab samples (diarrhea samples), and 47 bloody stool or rectal swab samples (bloody stool samples) (Table 1). Fresh stool and rectal samples were collected on transport agar (Seed Swab γ 1go; Eiken Chemical Co., Tokyo, Japan) using the swab included in the kit. Samples were kept at room temperature and were inoculated onto agar within 24 h.

**Table 1:** Number of fecal and rectal samples used in the survey.

	Sex		
Status of samples	Male	Female	Total
Clinically healthy	32	66	98
Diarrhea	36	49	85
Bloody stool	24	23	47

#### Isolation and identification of EPEC and other intestinal pathogens

Samples were inoculated on DHL agar (Eiken Chemical Co.) for detection of *E*. *coli* (including EPEC) and *Salmonella* spp., while SS agar (Becton Dickinson, Sparks, MD, USA) was used for detection of *Salmonella* spp., *Shigella* spp., and *Yersinia pseudotuberculosis*. Inoculated DHL agar plates were incubated under aerobic conditions at 37°C for 24 h (DHL agar) or 48 h (SS agar). For all suspected colonies of each pathogen (with the exception of *E*. *coli*), Gram staining as well as catalase, oxidase, and biochemical tests were carried out using commercially available kits (ID test EB20; Nissui Pharmaceutical Co., Tokyo, Japan). Antimicrobial susceptibility was tested using a commercially available kit (ATB VET; bioMérieux, Lyon, France). *E*. *coli* isolates were examined after confirming the presence of the *eae* gene with a secondary test (see below). For EPEC detection, 10 typical *E*. *coli* colonies on DHL agar were selected and mixed with 200 μl sterilized phosphate-buffered saline (PBS) to obtain a mixed colony suspension as a sample for the screening test. A portion of this suspension was inoculated on Heart Infusion Agar (Eiken Chemical Co.) and incubated under aerobic conditions at 37°C for 24 h for the secondary test. The suspensions were also tested for the presence of the VT1, VT2, LT, ST, *eae*, and *bfp* genes by PCR in the screening test. VT1, VT2, LT, and ST genes were detected with a commercially available PCR kit (PowerCheck Diarrheal *E*. *coli* 4-plex Detection kit I; Kogene Biotech Co., Seoul, Korea), while *eae* and *bfp* were detected as previously described [7,8]. When mixed colony suspensions showed positive results for the *eae* gene, each isolated bacterial colony on the Heart Infusion Agar was examined by PCR in a secondary test.

#### EPEC serotyping and O antigen genotyping

All EPEC isolates were serotyped by an agglutination test using a commercially available antisera kit (Antisera for Pathogenic *Escherichia coli*; Denka Seiken Co., Tokyo, Japan). O antigen genotyping was carried out for three selected isolates as previously described [9] at Miyazaki University.

### Experimental infection study

#### Animals and rearing environment

Male common marmosets (n = 10; 11–12 months old) were obtained from a commercial vendor (CLEA Japan, Tokyo, Japan). The animals weighed 276–320 g at the time of infection. Animals were housed individually in stainless steel cages (409 × 610 × 1578 mm). The cages were positioned facing each other at a distance of 1.7 m to allow the animals to communicate visually and vocally. Animals were reared individually in order to obtain exact results in terms of fecal condition and to prevent secondary infection from cage-mates. Animals were provided wooden perches and a resting place made of wood as environmental enrichment, and were fed a balanced commercial primate diet (CMS-1M; CLEA Japan) supplemented with ascorbic acid (Nacalai Tesque, Kyoto, Japan), vitamins A, D3, and E (Duphasol AE3D; Kyoritsu Seiyaku, Tokyo, Japan), and honey (Nihonhatimitsu, Gifu, Japan) and tap water *ad libitum*. The food was moistened with hot water to vary food texture. In addition to the standard diet, animals were fed with sponge cake or biscuits by humans. The room was maintained at constant temperature (27°C) and relative humidity (40%–60%) on a 12:12-h light/dark cycle. Therapeutic treatment with antibiotics and/or analgesics was not provided, because such a treatment would have affected the experimental results. All animals were clinically healthy and confirmed free from *Salmonella* spp., *Shigella* spp., EPEC, and *Yersinia pseudotuberculosis* by culture tests of fecal samples.

#### Bacterial strains

EPEC R811 used for the experimental infection study was isolated from the bloody stool sample of a common marmoset and identified as EPEC based on biochemical features and by PCR (positive for the *eae* gene and negative for VT1, VT2, LT, ST, and *bfp* genes). Biochemical characteristics and antimicrobial susceptibility of the strain are shown in Table 2.

**Table 2:** Biochemical characteristics and antimicrobial susceptibility of the strain of *Escherichia coli* used in this study.

Characteristics		Antimicrobial susceptibility^a^	
Oxidase	−	Penicillin	R
Catalase	+	Amoxicillin	R
Nitrate reduction	−	Amoxicillin-Clavulanate	R
Esculin hydrolysis	−	Oxacillin	R
Phenylpyruvic acid reaction	−	Cefalotine	R
Indole production	+	Cefoperazone	S
Voges–Proskauer test	−	Streptomycin	R
Citrate utilization	−	Spectinomycin	R
Lysine decarboxylase	+	Kanamycin	R
Arginine dihydrolase	−	Gentamycin	R
Ornithine decarboxylase	+	Apramycin	S
Orthonitrophenyl galactoside test	−	Chloramphenicol	S
Urease	−	Tetracycline	S
Malonic acid utilization	−	Doxycycline	S
Acid source		Erythromycin	R
Adonit	−	Lincomycin	R
Inositol	−	Pristinamycin	R
Raffinose	−	Tylosin	R
Rhamnose	+	Colistin	S
Sorbitol	+	Cotrimoxazole	S
Sucrose	−	Sulfamethizole	R
Mannitol	+	Flumequine	S
Arabinose	+	Oxolinic acid	S
		Enrofloxacin	S
		Nitrofurantoin	S
		Fusidic acid	R
		Rifampicin	R
		Metronidazole	R

^a^ R: Resistant, S: Sensitive

#### Study design of experimental infection

Two experimental groups (high and low concentrations; n = 4 animals each) and one negative control group (n = 2 animals) were used in this study. The number of animals was selected based on a previous experimental infection study of common marmosets and was determined according to the number of cages in the biosafety level (BSL) 2 experimental room at our institute [10].

Animals in the experimental group were perorally inoculated with 2 ml bacterial suspension adjusted to 5 × 10^8^ (high concentration) or 5 × 10^4^ (low concentration) CFU/ ml with sterile saline. These concentrations were determined based on previous experimental EPEC infection study in rhesus monkeys [11]. Animals in the negative control group were inoculated with 2 ml sterile saline. Two animals in each experimental group were euthanized 3 days post-inoculation (DPI), while the remaining two animals in each group were euthanized at 14 DPI. Animals in the negative control group were autopsied at 14 DPI. Animals were euthanized by exsanguination from the abdominal portion of the vena cava under ketamine (30 mg/kg) and isoflurane anesthesia. The body weight of each animal was measured daily.

#### Re-isolation of bacterial strains

Bacterial strains were isolated from blood, duodenum contents, the cecum at autopsy, and daily from feces. Samples (except those from blood) were streaked directly onto DHL agar; 100 μl of blood was inoculated on DHL agar. Bacterial isolates were identified as described above.

#### Histopathological examination

Esophagus, heart, lungs, mesenteric lymph nodes, spleen, stomach, small and large intestines, liver, gallbladder, pancreas, kidney, duodenum, cecum, and rectum were examined histologically. Tissue samples were fixed in 10% neutral buffered formalin, embedded in paraffin, and stained with hematoxylin and eosin (HE) and toluidine blue and subjected to Gram staining according to standard procedures.

#### Statistical analysis

Pearson’s χ^2^ test was used to compare EPEC isolation rates among clinically healthy, diarrhea, and bloody stool samples. Fisher’s exact test with Holm’s correction for multiple tests [12] was used to compare EPEC isolation rates between any pair of proportions for more than two groups, with adjustment for type I errors in multiple comparisons. Data were analyzed using R software, release 3.2.1 (R Foundation for Statistical Computing, Vienna, Austria. http://www.R-project.org). Significance was determined at P < 0.01.

#### Ethics statement

This study was carried out in strict accordance with the Regulations for Animal Experimentation of the CIEA based on the Guidelines for Proper Conduct of Animal Experiments (Science Council of Japan, 2006). The protocol was approved by the Institutional Animal Care and Use Committee (IACUC) for Animal Experimentation of the CIEA (permit no. 14007A). All animals in the study were reared in a BSL2 room used exclusively for experimental infection studies had free access to food and water, and were checked at least twice daily. All efforts were made to minimize painful procedures; when these were necessary, an attending veterinarian was consulted. Following the development of clinical signs, animals were checked multiple times daily. When clinical observations and scores reached defined levels based on the approved IACUC protocol, animals were sacrificed under deep anesthesia to minimize pain and distress. When animals became moribund or at the end of study, they were humanely euthanized as described above in accordance with current American Veterinary Medical Association Guidelines for Euthanasia of Animals.

## Results

### EPEC prevalence in common marmosets

A total of 74 stool or rectal swab samples were positive for EPEC. EPEC was detected in 10 of 98 clinically healthy samples (10.2%), 17 of 85 diarrhea samples (20%), and all 47 bloody stool samples (100%) (Table 3). *Salmonella* spp., *Shigella* spp., and *Y*. *pseudotuberculosis* were not detected in any of the samples.

**Table 3:** Results of isolation of enteropathogenic *Escherichia coli* from this survey.

	No. of positive samples/no. of samples tested			
Samples	Male	Female	Total	Total positive rate (%)
Clinically healthy	4/ 32	6/ 66	10/ 98	10.2
Diarrhea	6/ 36	11/ 49	17/ 85	20
Bloody stool	24/ 24	23/ 23	47/ 47	100

All 74 EPEC isolates were identified as *E*. *coli* using a commercially available biochemical test kit. The isolates were positive for the *eae* gene and negative for VT1, VT2, LT, ST, and *bfp* genes by PCR. The serotypes of all isolates were undeterminable by the antisera kit. Three selected isolates were determined to be of the O85 genotype.

### Experimental infection study

#### Clinical symptoms and re-isolation of EPEC strains

**Fig 1:**
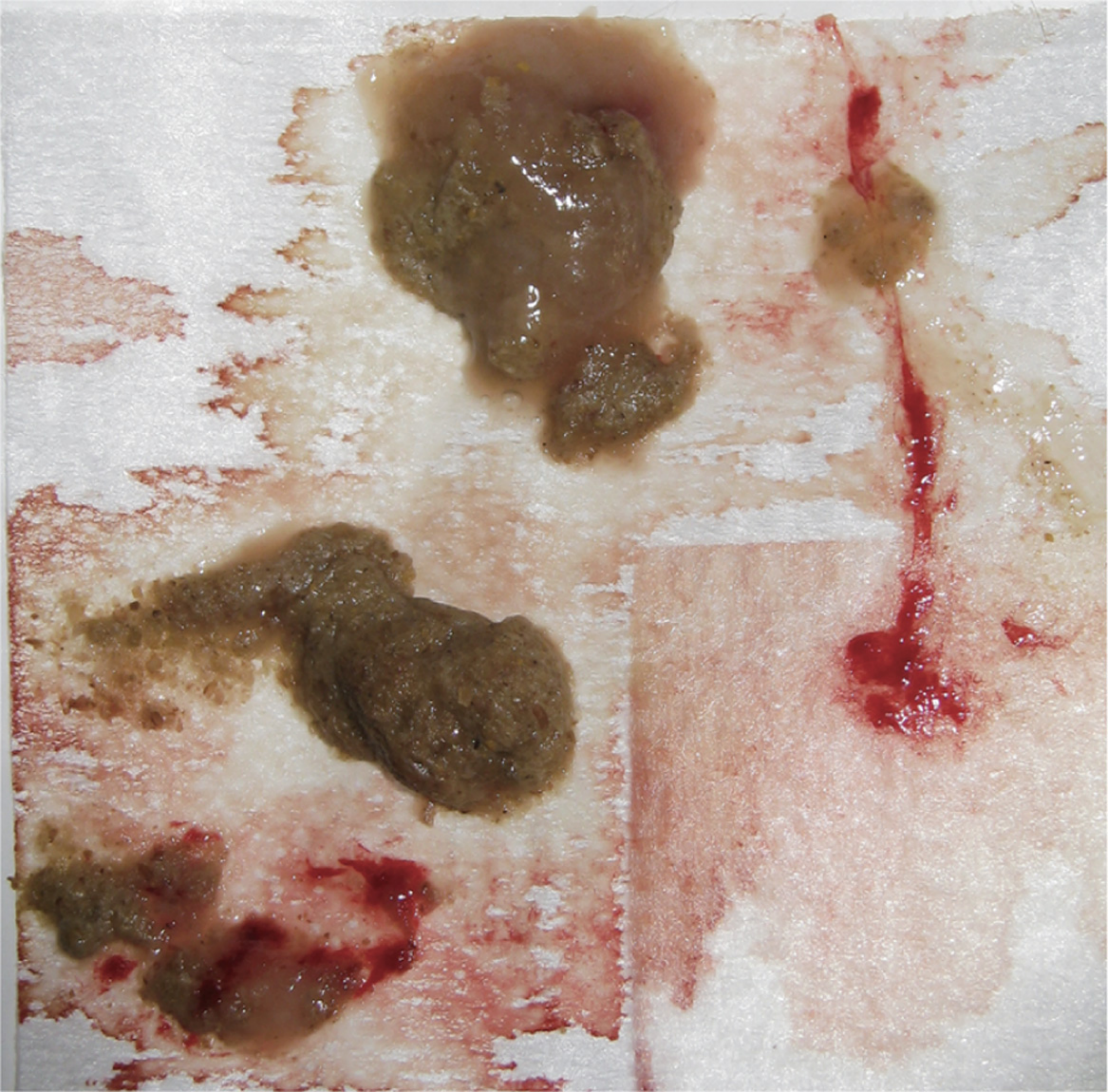
Bloody stool from common marmoset experimentally infected with Enteropathogenic *E*. *coli* (ID no. 2 in the high-concentration group) at 2 days post-inoculation.

**Fig 2:**
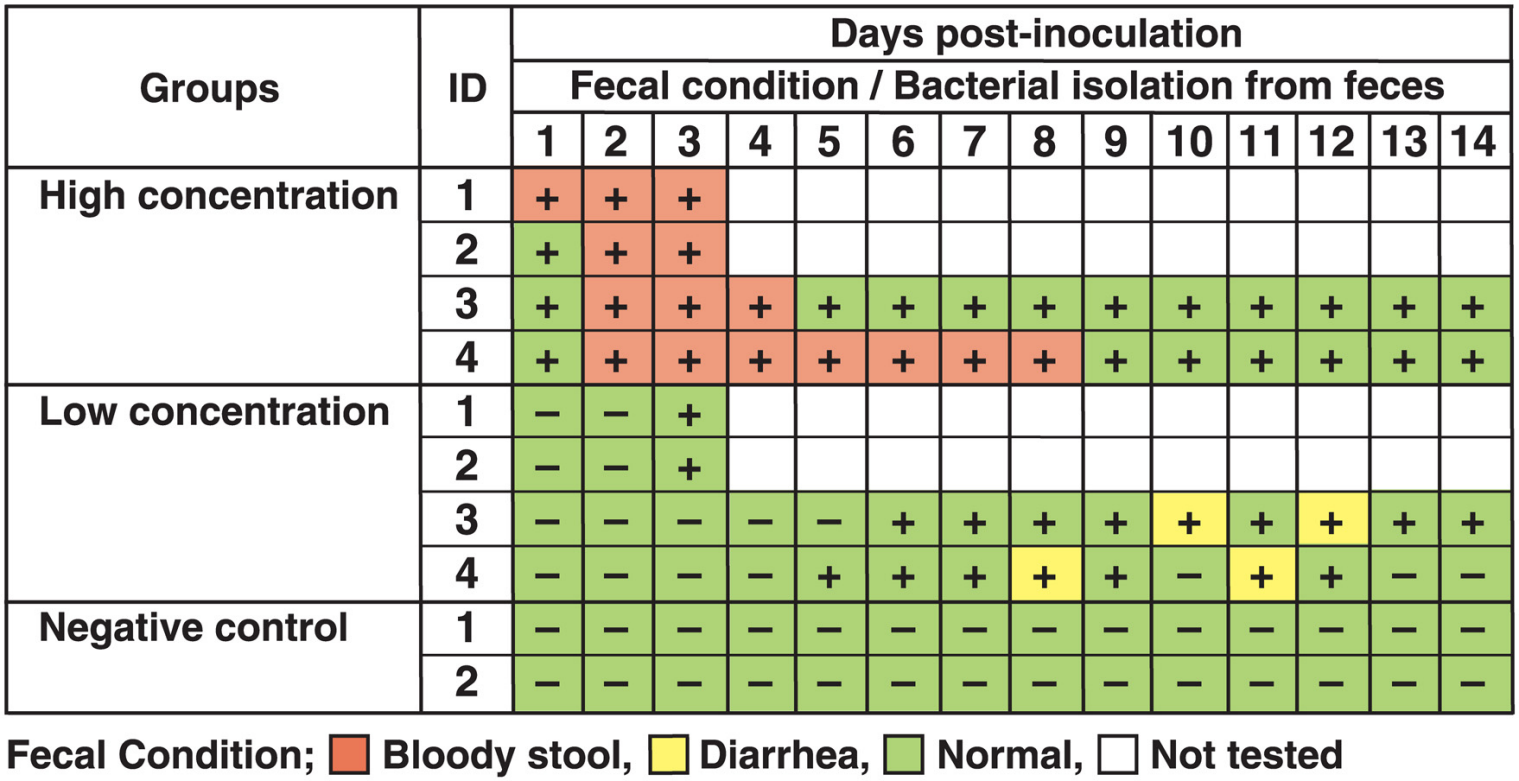
Relationship between fecal condition and isolation of enteropathogenic *Escherichia coli* (EPEC) in the experimental study. +, positive for EPEC; -, negative for EPEC.

Two animals in the high-concentration group showed slight lethargy at 1 DPI and hematochezia at 1 and 2 DPI (Fig 1), which lasted until autopsy at 3 DPI. The remaining two animals in this group showed hematochezia without lethargy at 2 DPI, which lasted until 4 and 8 DPI. The EPEC strain was re-isolated only from feces and the cecum (at autopsy) in all animals in this group during the experimental period. In the low-concentration group, two animals autopsied at 3 DPI showed no clinical symptoms. The remaining two animals exhibited diarrhea in the latter half of the experimental period (at 8 and 11 DPI for one animal and at 10 and 12 DPI for the other). In the two animals autopsied at 3 DPI, the EPEC strain was not re-isolated from any regions tested except from feces and cecum. The EPEC strain was re-isolated only from feces and cecum (a single animal at autopsy) of the remaining two animals from 6–14 DPI and from 5–12 DPI but not at 10 DPI. No clinical symptoms were observed in the negative control group, and the EPEC strain was not re-isolated from these animals. The results are summarized in Fig 2.

### Autopsy and histopathology

**Fig 3:**
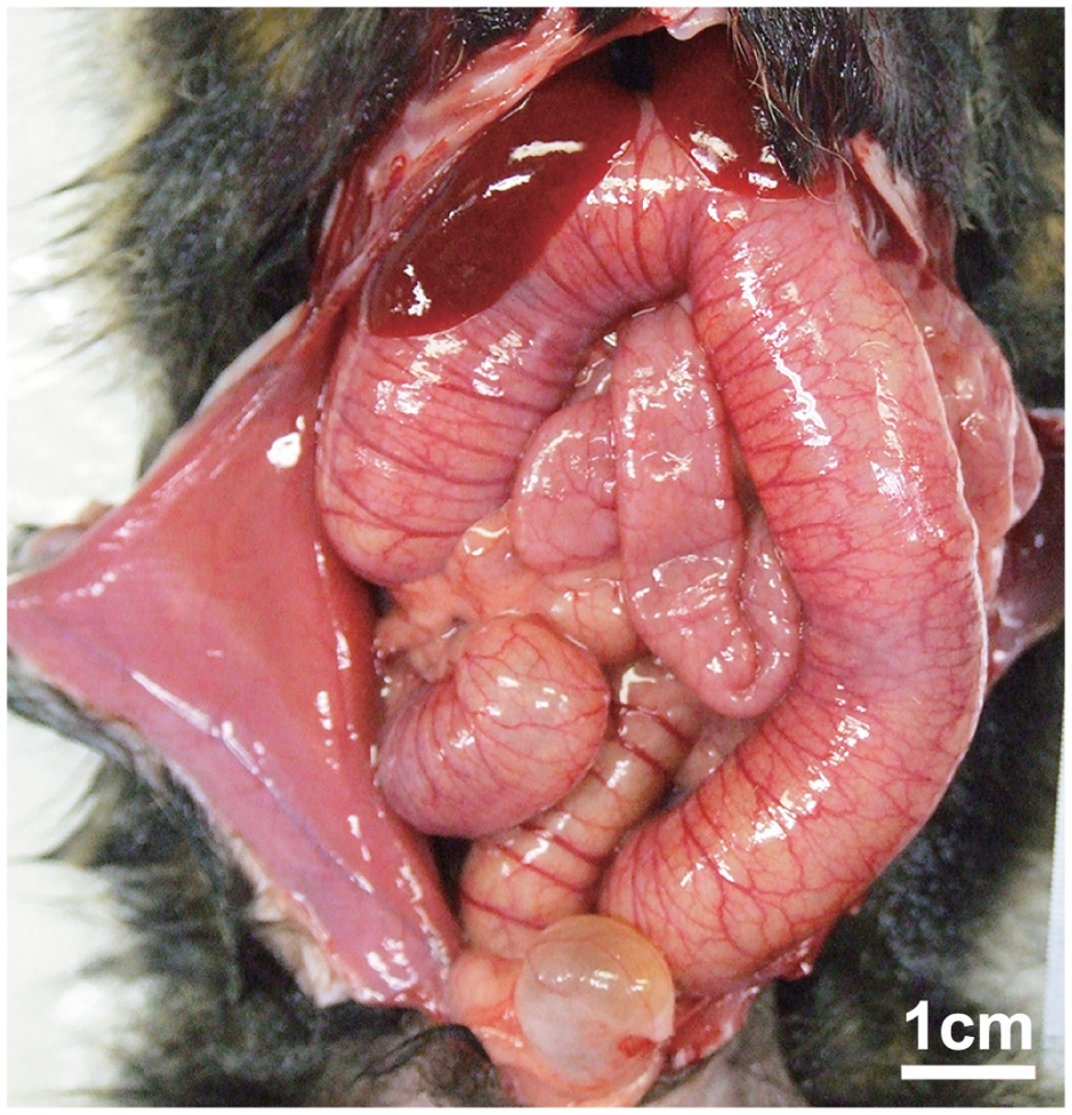
Hyperemia and atony of large intestines in common marmosets experimentally infected with Enteropathogenic *E*. *coli* (ID no. 2 in the high-concentration group) at 3 days post-inoculation.

**Fig 4:**
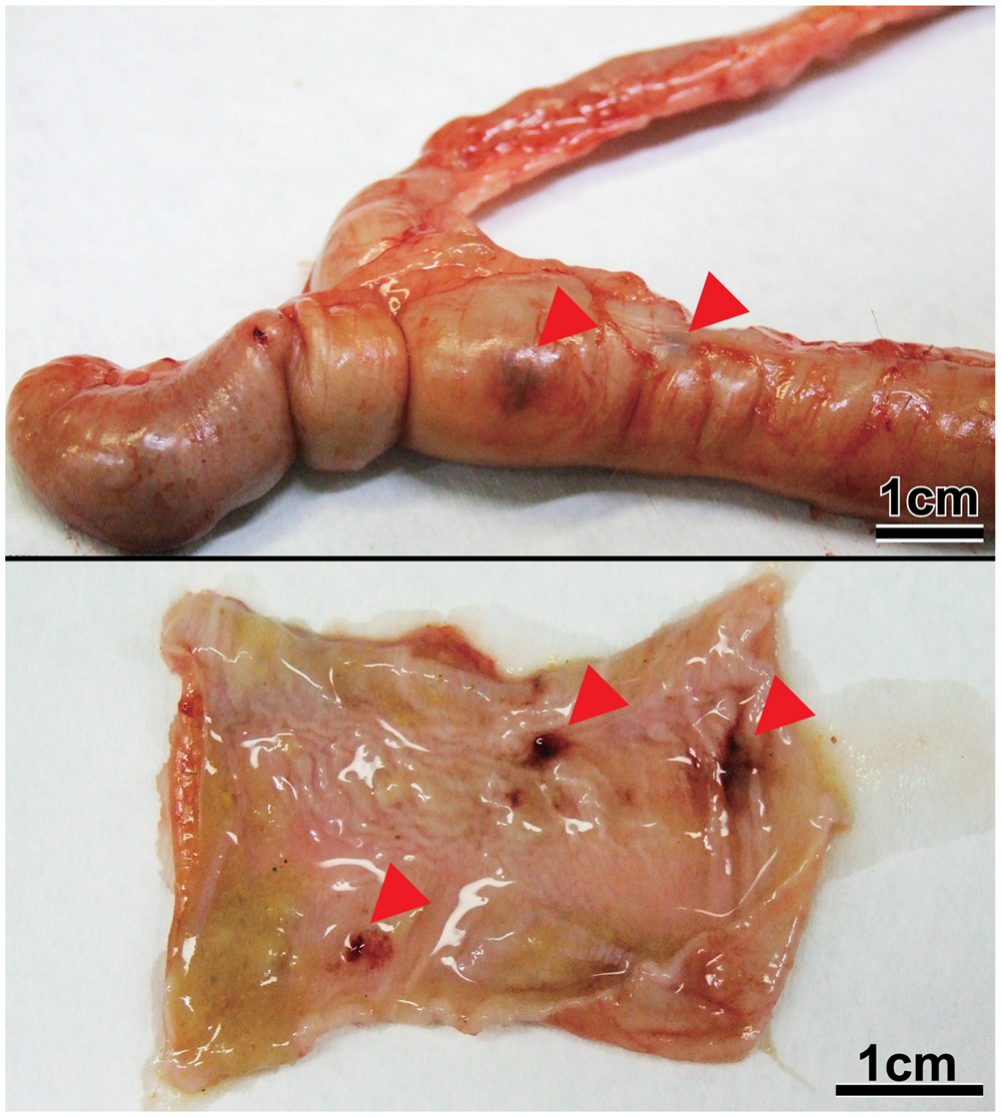
Petechia of colon mucosa in common marmosets experimentally infected with Enteropathogenic *E*. *coli* (ID no. 2 in the high-concentration group) at 3 days post-inoculation (top, petechia from the serosal side; bottom, hemorrhage from the mucosal side).

**Fig 5:**
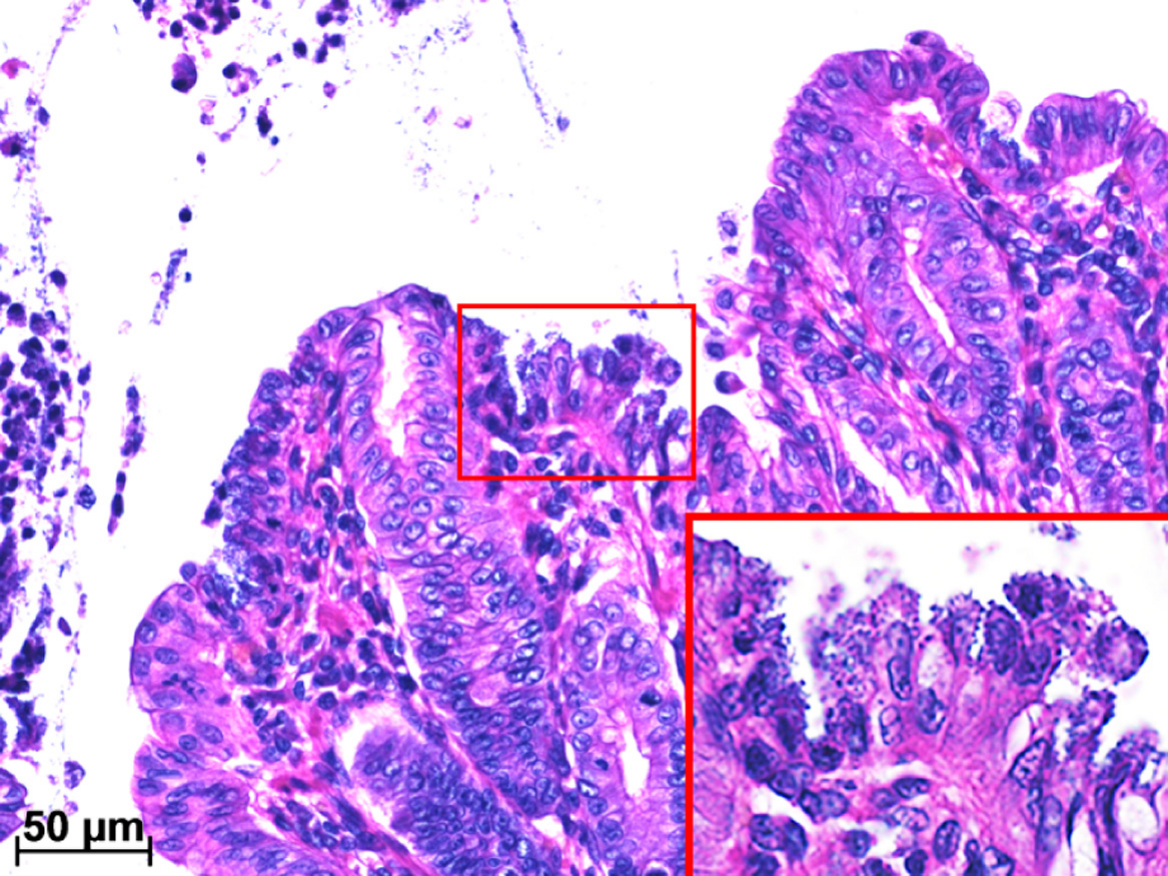
Photomicrograph of a section of cecum from common marmoset no. 1 in the high-concentration group at 3 days post-inoculation. Bacilli were attached to epithelial apical membranes (inset) and desquamated epithelial cells, as visualized by H-E staining.

Two animals in the high-concentration group exhibiting slight lethargy and two animals in the low-concentration group without clinical symptoms were autopsied at 3 DPI. Slight ascites, enlargement of mesenteric lymph nodes, swollen spleen, hyperemia and atony of large intestine, and petechia of colon mucosa were observed in both animals in the high-concentration group (Figs 3 and 4). A histopathological examination revealed lymphoid hyperplasia in the mesenteric lymph nodes and spleen. In the cecum, gram-negative bacilli were attached to epithelial apical membranes and desquamated epithelial cells (Fig 5). There were no histological abnormalities in other organs. No gross lesions were observed in either animal in the low-concentration group. At 14 DPI, no gross lesions were observed in any of the animals examined (two each in the high- and low-concentration and negative control groups), except for enlargement of the mesenteric lymph nodes in the high-concentration group. These lesions were identified as lymphoid hyperplasia.

### Statistical analysis

Based on Pearson’s χ^2^ test, the three groups differed in terms of EPEC isolation rate (P < 0.01). Using the Holm method, there were significant differences in the EPEC isolation rate between the bloody stool sample group and the other two groups (adjusted P < 0.01 in both cases by Fisher’s exact test). There was no difference in EPEC isolation rate between clinically healthy and diarrhea samples (adjusted P = 0.09 by Fisher’s exact test).

## Discussion

There was a significant difference between sample status and EPEC presence. The EPEC-positive rate was higher in bloody stool samples than in healthy or diarrhea samples. The pathologic potential of EPEC in nonhuman primates has been previously reported. In one study of 18 nonhuman primates including nine common marmosets, the isolation ratio of EPEC from diarrhea and/or enteritis (47%) was higher than from apparently healthy animals (27%) [13]. In another study, EPEC was associated with hemorrhagic typhlocolitis in three cases of necropsy of common marmosets during an outbreak at the experimental facility [4]. The results of our survey using a relatively large number of samples are in accordance with this earlier work, and demonstrate the pathogenic potential of EPEC in hematochezia of common marmosets.

In a previous report, *E*. *coli* serogroup O26 was the causative strain of a hemorrhagic typhlocolitis outbreak in common marmosets [4] and was associated with diarrhea in a nonhuman primate (*Saguinus fuscicollis*) [13]. The isolates in the present study belonged to serogroup O85, suggesting that various serogroups of EPEC strains are responsible for hemorrhagic typhlocolitis in common marmosets. Although previous studies have shown an association between EPEC and hemorrhagic typhlocolitis in these animals, most employed retrospective approaches such as necropsy [4] and bacterial cultures of isolates [13].

The actual pathogenicity of EPEC in common marmosets is poorly documented. We therefore carried out experimental infection of common marmosets with EPEC. We observed acute hematochezia in all animals in the high-concentration group, although symptoms disappeared between 5 and 9 DPI in animals that were monitored until 14 DPI. On the other hand, no hematochezia was observed in the low-concentration group, which was monitored until 14 DPI. Two animals in this group had diarrhea twice from 8–12 DPI. These results indicate that the onset of hematochezia depends on the quantity of EPEC cells to which an animal is exposed.

EPEC was recovered from all animals in the high-concentration group throughout the experimental period. On the other hand, EPEC was recovered from two animals at 3 DPI and two at 5 and 6 DPI in the low-concentration group; of the latter, EPEC disappeared in one animal after 13 DPI, suggesting that it can cause continuous infection in common marmosets.

The histopathological examination revealed attachment of gram-negative bacilli to epithelial apical membranes and desquamation of cecal epithelial cells in animals in the high-concentration group at 3 DPI (Fig 5). This is similar to previously reported observations [4]. In human disease, EPEC is known to cause acute, watery diarrhea but not hematochezia, especially in infants [8,14]. In our study, hematochezia caused by desquamation of cecal epithelial cells was observed in animals as a main symptom, but differed from that in humans. The presence of an unidentified virulence factor in *E*. *coli* strains derived from hemorrhagic typhlocolitis in common marmosets has been proposed to explain the difference in clinical symptoms between the two species [4]. Recently, our team identified by whole-genome sequencing genes related to pathogenicity such as RTX toxin (*rtxA1*), hemolysin E (*ClyA*), and Shigella enterotoxin 2 (*shET2*) in the EPEC R811 strain used in the present experimental study [15]. Although further research is necessary, these pathogenic factors may be responsible for causing hemorrhagic typhlocolitis in common marmosets.

In conclusion, we demonstrated a high prevalence of EPEC infection with hematochezia in common marmosets using a relatively large number of samples, and induced hemorrhagic typhlocolitis in marmosets. This is the first study that has reproduced this disease in these animals by experimental EPEC infection. Our findings provide insight into the causes of diarrhea in common marmosets as well as an experimental model for studying this disease.
